# Effects of thinning and understory removal on soil phosphorus fractions in subtropical pine plantations

**DOI:** 10.3389/fpls.2024.1416852

**Published:** 2024-06-25

**Authors:** Zunji Jian, Lixiong Zeng, Lei Lei, Changfu Liu, Yafei Shen, Jiajia Zhang, Wenfa Xiao, Mai-He Li

**Affiliations:** ^1^ Key Laboratory of Forest Ecology and Environment of National Forestry and Grassland Administration, Ecology and Nature Conservation Institute, Chinese Academy of Forestry, Beijing, China; ^2^ Co-Innovation Center for Sustainable Forestry in Southern China, Nanjing Forestry University, Nanjing, China; ^3^ Forest Dynamics, Swiss Federal Institute for Forest, Snow and Landscape Research WSL, Birmensdorf, Switzerland; ^4^ Key Laboratory of Geographical Processes and Ecological Security in Changbai Mountains, Ministry of Education, School of Geographical Sciences, Northeast Normal University, Changchun, China; ^5^ School of Life Science, Hebei University, Baoding, China

**Keywords:** understory removal, selective logging, Hedley’s P fraction, soil P dynamic, P-deficient plantation, Pinus massoniana

## Abstract

Forest management changes the physical environments and nutrient dynamics and then regulates the forest productivity. Soil phosphorus (P) availability is critical for productivity in tropical and subtropical forests. However, it was still poorly understood how soil P content and fraction respond to various forest management practices in these regions. Here, we measured the soil total P, available P, and Hedley’s P fractions, including inorganic and organic P (Pi and Po), in subtropical pine plantations treated with understory removal (UR), non-dominant species thinning (NDST) and dominant species thinning (DST) after nine years. Compared to plantations without management (CK), treatments such as UR, NDST, and DST decreased soil total P at 0–10 cm and soil available P at 0–10 cm and 10–20 cm. Increases in resin-Pi, NaOH-Pi, and C.HCl-Pi resulted in a higher total Pi in 0–10 cm (*p* < 0.05) in treated plots (UR, NDST, and DST) than in CK plots. UR, NDST, and DST treatments increased NaHCO_3_-Po and NaOH-Po (*p* < 0.05) but decreased C.HCl-Po at a depth of 0–10 cm. Regardless of management treatments, soil total P, available P, and P fractions in 0–10 cm showed higher contents than those in 10–20 cm. There were positive relationships between total P and total Po (*p* < 0.01) and between available P and total Pi. There were also positive relationships between total P, available P, NaHCO_3_-Pi, and NaOH-Pi (*p* < 0.05). In conclusion, forest management such as UR, NDST, and DST decreased soil total P and available P, and transforming soil P fractions to available P will meet the P demand following management in the pine plantations of subtropical China.

## Introduction

Due to phosphorus (P) leaching losses and recalcitrant P fraction formation (Walker and Syers, 1976), low soil P availability often limits productivity in forest ecosystems, especially in tropical and subtropical regions (Vitousek et al., 2010; Reichert et al., 2022). Stand-level management practices, such as understory removal (UR) and selective thinning, improve plant growth by changing the physical environments (Trentini et al., 2017; Giuggiola et al., 2018) and nutrient cycles (Rocha et al., 2019; Qiu et al., 2020). Therefore, clarifying the responses of soil P pools to UR and thinning practices is essential in developing management strategies to improve forest productivity (Noormets et al., 2015).

Although the thinning effects on soil P pools differed in a specific forest, the global meta-analysis found that selective thinning generally increases soil total P and available P contents (Zhou et al., 2021; Zhang et al., 2023). In tropical rain forests, thinning can decrease soil total P and organic P (Imai et al., 2012; Lontsi et al., 2019). However, thinning was found not to affect soil total P in the subtropical spruce forests (Hu et al., 2016) or to decrease soil available P in subtropical coniferous mixed forests (Zhou et al., 2015). Thinning treatment increased soil available P in temperate spruce and larch plantations (Zhou et al., 2019; Zhao et al., 2023) but not in a temperate pine forest (Kaye et al., 2005). Moreover, a meta-analysis pointed out that UR decreases soil total P but increases soil available P (Zhang et al., 2022a). Usually, due to differences in plant growth and their P demand for various species (Reichert et al., 2022), soil total and available P dynamics under forest management may differ in various forests, and thus, further studies are needed.

Soil P includes inorganic (Pi) and organic (Po) forms, which are divided into multiple fractions (Hedley et al., 1982; Tiessen and Moir, 1993). Although soil total P stock is always larger than vegetation P stock (Imai et al., 2010), it is challenging to meet the P demand for plant growth due to differences in the availability of multiple P forms (Hedley et al., 1982). The P fractions extracted by Hedley’s method have been used to explore the environmental and management effects on soil P pools (Negassa and Leinweber, 2009) and provide crucial information on soil P dynamics (Liu et al., 2021). Previous studies found that variations in soil P fractions varied with thinning intensity, forest type, soil type, and geographic location (Ye et al., 2018; Liu et al., 2018b). UR treatment only significantly reduced residual-P concentration (Zhang et al., 2022b). To our knowledge, however, which soil P fractions are available as potentially usable P for plants after forest management is unknown.

Here, we collected soil samples in pine plantations treated with UR and selective thinning after nine years in subtropical China. We measured the total P, available P, and Hedley’s P fractions, including Pi and Po, to examine the differences between management treatments. Our previous study observed a significant positive effect of soil P availability on stand productivity in pine plantations (Jian et al., 2022a). We therefore hypothesize that forest management decreases soil total P and available P contents (Hypothesis 1) due to the increased plant growth after forest management (Lei et al., 2021). Because soil microbes often accelerate the transformation of Po to Pi (Fan et al., 2018; Liu et al., 2021), we expect that soil Pi rather than Po fractions will increase after forest management (Hypothesis 2). To meet increased P demand for plant growth following forest management (**Table 1**), we predict that soil available P increases with decreasing soil Po and residual-P fractions (Hypothesis 3). A previous study showed that ectomycorrhizal (ECM) and arbuscular mycorrhizal (AM) trees responded differently to Pi addition in tropical and subtropical forests (Liu et al., 2018a). Therefore, in the selected pine (an ECM tree) plantation, our results will provide new insights into understanding soil P dynamics under forest management.

**Table 1:** Information on the selected plantations after treatments in September 2013.

Variable	Control (CK)	Understory removal (UR)	Non-dominant species thinning (NDST)	Dominant species thinning (DST)
Topographical features				
Altitude (m)	1225 ± 8	1240 ± 3	1200 ± 4	1226 ± 6
Slope (°)	34	35	33	33
Aspect	Northwest	Northwest	Northwest	Northwest
Stand characteristics ^†^				
Average DBH (cm)	11.10 ± 0.37	12.50 ± 0.43	17.74 ± 0.56	9.36 ± 0.30
Average height (m)	8.33 ± 0.14	8.91 ± 0.16	12.35 ± 0.24	8.54 ± 0.58
ΔDBH (cm) ^§^	0.96 ± 0.09	1.10 ± 0.07	1.12 ± 0.06	1.04 ± 0.12

^†^Values are shown as mean ± standard errors.

^§^ΔDBH represents the increment of diameter at breast height (1.3 m) for the remaining trees between 2013 and 2016 (Lei et al., 2021).

## Materials and methods

### Site description

The study was conducted at Jiulingtou Forest Farm (30°59′N, 110°47′E) in subtropical China. The site is characterized by a typical humid monsoon climate, with a mean annual temperature of 16.9°C and a mean annual precipitation between 1000 mm and 1250 mm (Lei et al., 2018). The zonal soil type is yellow-brown soil (Cambisols, Shi et al., 2010) with a 1.0–1.2 m depth. Reforestation in the study region was widely implemented to alleviate land degradation resulting from deforestation of climax vegetation.

The forest management experiment was performed in an aerially seeded *Pinus massoniana* forest established in the 1970s. At the beginning of the forest management experiment, the stand density was approximately 1700 stems per hectare (Lei et al., 2018; Shen et al., 2018), and the species composition mainly included coexisting trees (e.g., *Toxicodendron vernicifluum*, *Betula luminifera*, and *Cunninghamia lanceolataand*) and understory shrubs (e.g., *Pyracantha fortuneana*, *Litsea pungens*, and *Lespedeza bicolour*). In the early stage of forest management, soil total P is between 0.20 g·kg^-1^ and 0.24 g·kg^-1^ in this pine plantation (Lei et al., 2018). These soil total P contents had been categorized as very low in tropical and subtropical regions (Reichert et al., 2022). In other words, the pine plantations investigated in this study represent a P-deficient ecosystem to some extent.

### Experimental design

In September 2013, four experiment plots (20 m × 20 m) were set within three large pine plantations (Lei et al., 2018). One treatment for one plot in each plantation randomly put the following four practices. (1) Control (CK): the forest remains in its original state, except for slight disturbances (e.g., sampling) during the investigation. (2) Understory removal (UR): understory shrubs were removed yearly to reduce competition with overstory vegetation. (3) Non-dominant species thinning (NDST): the non-dominant species whose DBH is more than 5 cm were reduced by 15% (the basal area calculated) to reduce competition with dominant species. (4) Dominant species thinning (DST): the pine trees with a DBH of more than 17.9 cm were reduced by 75% (the basal area calculated) to reduce competition with other trees. All forest management practices have not treated any roots, living herbs, and floor litters (Lei et al., 2018) and significantly enhanced the annual increment of DBH for the remaining trees (**Table 1**).

### Soil sampling and analyses

Mineral soils at 0–10 cm and 10–20 cm were randomly sampled from nine points using a soil auger in July 2022. The soil samples in each plot were manually mixed and air-dried indoors to pass through a 2-mm sieve. Total P and available P contents were measured by plasma emission spectroscopy (IRIS Intrepid II XSP, Thermo Fisher Scientific, USA) following digestion with NHO_3_-HClO_4_-HF solution and using a continuous flow analyzer (Analytical AA3 Auto Analyser, SEAL, Germany) after extraction with HCl-H_2_SO_4_ solution, respectively (Jian et al., 2022b). The P fractions, including resin-Pi, NaHCO_3_-Pi, NaHCO_3_-Po, NaOH-Pi, NaOH-Po, HCl-Pi, C.HCl-Pi, C.HCl-Po, and residual-P, were measured by Hedley’s method (Hedley et al., 1982) and its modification (Tiessen and Moir, 1993).

### Statistical analysis

Data were tested to meet the normality requirements (Kolmogorow-Smirnow test) and homogeneity (Bartlett test) and were logarithmically transformed when necessary for subsequent analysis. One-way analyses of variance (ANOVA) and the Tukey HSD test were used to determine the differences in soil P pools among forest management practices and soil layers. The correlations among soil P pools were examined using Pearson’s correlation analysis and general linear regression models. All analyses were implemented in R 4.3.0 (R Development Core Team, 2023).

## Results

### Soil total P and available P contents

**Figure 1:**
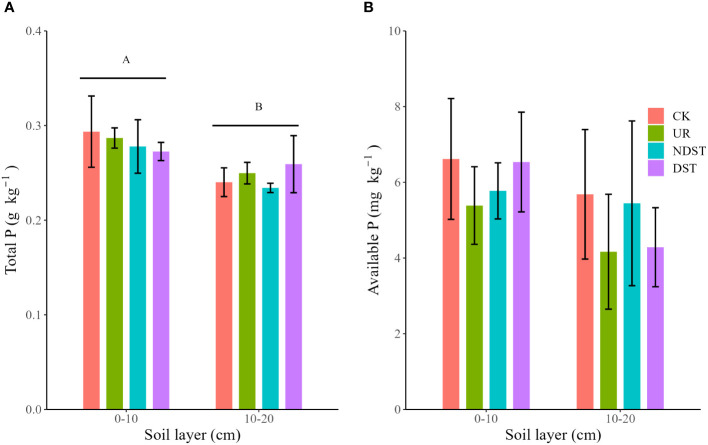
Comparison of total P **(A)** and available P **(B)** among forest management treatments. Values are means ± 1 standard deviation (*n* = 3). Different capital letters represent significant differences between soil layers (*p* < 0.05). CK, control; UR, understory removal; NDST, non-dominant species thinning; and DST, dominant species thinning.

Forest management treatments and their interactions with soil layers did not significantly affect soil total P and available P (**Supplementary Table S1**). Compared to CK, treatments such as UR, NDST, and DST declined soil total P (0–10 cm; **Figure 1A**) and soil available P (0–10 cm and 10–20 cm; **Figure 1B**) with no significant level. Regardless of management treatments, soil total P (0.28 ± 0.02 g·kg^-1^) in-depth 0–10 cm was greater (*p* < 0.001) than that in-depth 10–20cm (0.25 ± 0.02 g·kg^-1^) (**Figure 1A**).

### Soil P fractions

Forest management treatments, soil layers, and their interactions differently affected soil P fractions (*p* < 0.05; **Supplementary Table S1**). Soil C.HCl-Pi in 0–10 cm and C.HCl-Po in 10–20 cm were higher in NDST and DST plots than in CK plots (*p* < 0.05; **Table 2**). Overall, increased resin-Pi, NaOH-Pi and C.HCl-Pi in treatment plots resulted in higher total Pi in 0–10 cm in UR (51.53 ± 2.88 mg·kg^-1^), NDST (60.79 ± 6.49 mg kg^-1^) and DST (58.26 ± 4.31 mg·kg^-1^) than in CK plots (46.87 ± 2.44 mg·kg^-1^) (*p* < 0.05; **Table 2**). Compared to CK, treatments, including UR, NDST, and DST, increased NaHCO_3_-Po and NaOH-Po but decreased C.HCl-Po at 0–10 cm. Moreover, differences in P fractions between 0–10 cm and 10–20 cm varied in different treatments (**Table 2**).

**Table 2:** Comparison of soil P fractions among forest management treatments.

Soil layer (cm)	P fraction (mg·kg^-1^)	Control (CK)	Understory removal (UR)	Non-dominant species thinning (NDST)	Dominant species thinning (DST)
0–10	Resin-Pi	0.87 ± 0.24Aa	1.14 ± 0.30Aa	1.29 ± 0.34Aa	1.13 ± 0.26Aa
	NaHCO_3_-Pi	5.40 ± 0.31Aa	4.41 ± 0.91Aa	5.94 ± 1.72Aa	3.92 ± 0.58Aa
	NaHCO_3_-Po	10.78 ± 1.63Aa	14.57 ± 4.60Aa	14.79 ± 0.33Aa	12.60 ± 2.61Aa
	NaOH-Pi	16.94 ± 1.59Aa	19.66 ± 1.05Aa	20.22 ± 1.68Aa	20.51 ± 2.26Aa
	NaOH-Po	66.85 ± 2.72Aab	69.65 ± 2.42Aa	72.25 ± 2.51Aa	62.12 ± 4.25Ab
	HCl-Pi	2.95 ± 0.37Aa	1.92 ± 0.78Aa	3.32 ± 1.83Aa	2.61 ± 0.67Aa
	C.HCl-Pi	20.71 ± 3.21Ab	24.40 ± 4.21Aab	30.02 ± 2.27Aa	30.09 ± 3.33Aa
	C.HCl-Po	28.21 ± 10.93Aa	25.15 ± 5.36Aa	17.28 ± 3.94Aa	21.55 ± 2.00Aa
	Residual-P	50.24 ± 10.28Aa	58.61 ± 15.19Aa	62.50 ± 1.75Aa	66.21 ± 2.61Aa
	Total Pi	46.87 ± 2.44Ac	51.53 ± 2.88Abc	60.79 ± 6.49Aa	58.26 ± 4.31Aab
	Total Po	105.84 ± 6.90Aa	109.37 ± 10.58Aa	104.32 ± 2.42Aa	96.28 ± 6.68Aa
10–20	Resin-Pi	1.19 ± 0.09Aa	0.80 ± 0.21Ab	1.03 ± 0.11Aab	1.22 ± 0.03Aa
	NaHCO_3_-Pi	3.09 ± 1.38Ba	2.85 ± 0.17Ba	3.37 ± 1.24Aa	2.47 ± 0.75Aa
	NaHCO_3_-Po	10.98 ± 1.27Ba	9.27 ± 2.59Aa	7.45 ± 1.61Ba	14.12 ± 6.92Aa
	NaOH-Pi	14.38 ± 2.73Aa	13.71 ± 0.22Ba	14.42 ± 2.54Ba	13.89 ± 2.23Ba
	NaOH-Po	65.68 ± 6.71Aa	69.79 ± 1.55Aa	70.25 ± 6.63Ba	62.78 ± 9.91Aa
	HCl-Pi	1.52 ± 0.67Ba	2.03 ± 0.38Aa	1.93 ± 0.43Aa	1.96 ± 0.42Aa
	C.HCl-Pi	23.68 ± 2.11Aa	23.80 ± 2.06Aa	23.60 ± 2.81Ba	20.75 ± 3.72Ba
	C.HCl-Po	12.52 ± 1.35Ab	16.95 ± 4.92Aab	18.34 ± 1.07Aa	20.99 ± 3.33Aa
	Residual-P	62.59 ± 2.50Aab	54.46 ± 2.64Ab	64.64 ± 14.51Aab	75.72 ± 6.98Aa
	Total Pi	43.86 ± 4.54Aa	43.18 ± 1.87Aa	44.35 ± 3.80Ba	40.28 ± 5.37Ba
	Total Po	89.18 ± 6.21Ba	96.00 ± 7.27Aa	96.04 ± 8.34Aa	97.89 ± 11.17Aa

Values are means ± 1 standard deviation (n = 3). Different capital letters in the same column represent significant differences for the same variable between layers (p < 0.05), and different lowercase letters in the same row represent significant differences between treatments (p < 0.05). Pi, inorganic phosphorus; and Po, organic phosphorus.

### Relationships between soil P pools

**Figure 2:**
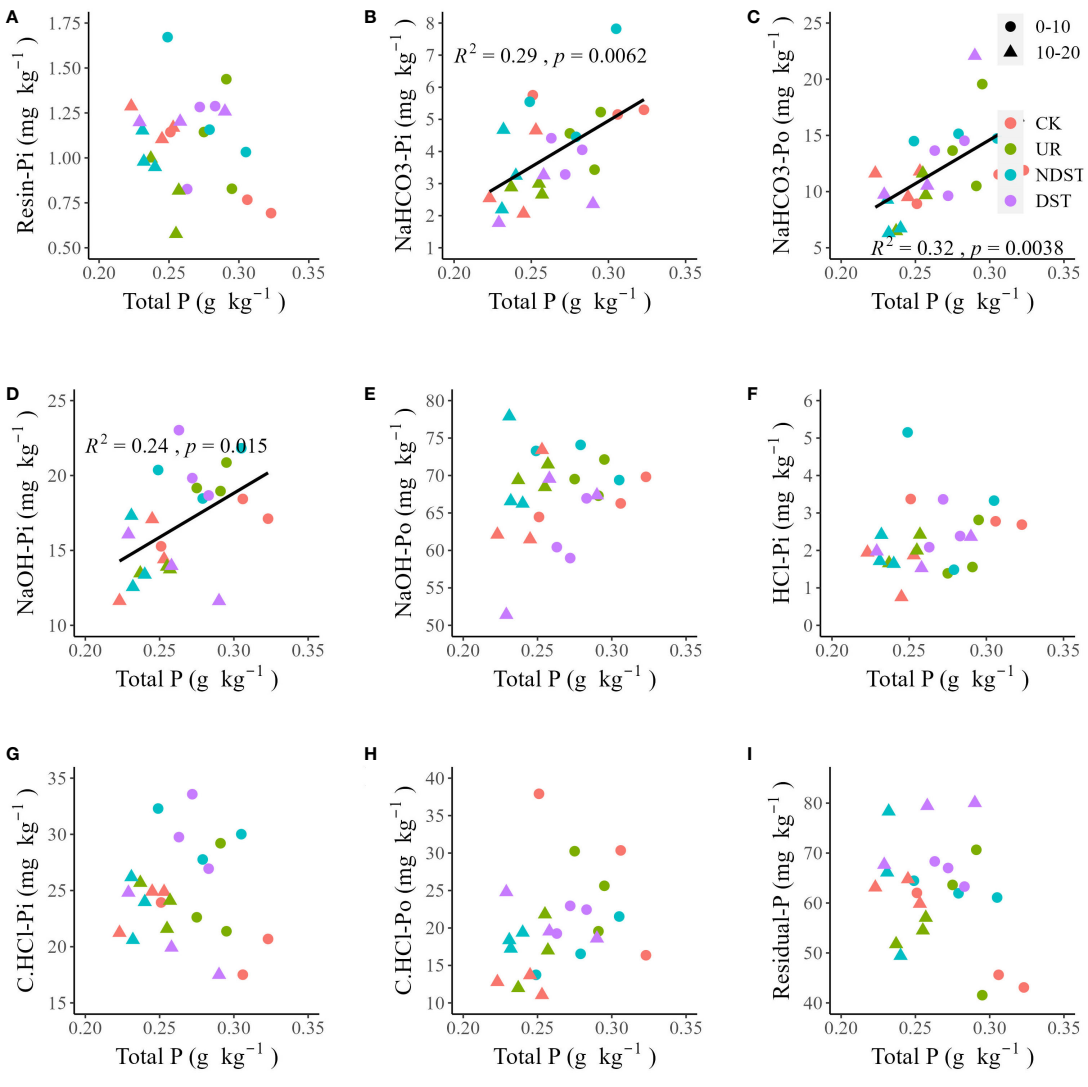
Correlations of soil total P with resin-Pi **(A)**, NaHCO_3_-Pi **(B)**, NaHCO_3_-Po **(C)**, NaOH-Pi **(D)**, NaOH-Po **(E)**, HCl-Pi **(F)**, C.HCl-Pi **(G)**, C.HCl-Po **(H)** and residual-P **(I)**. CK, control; UR, understory removal; NDST, non-dominant species thinning; DST, dominant species thinning; 0–10, 0–10 cm soil layer; 10–20, 10–20 cm soil layer; Pi, inorganic phosphorus; and Po, organic phosphorus.

**Figure 3:**
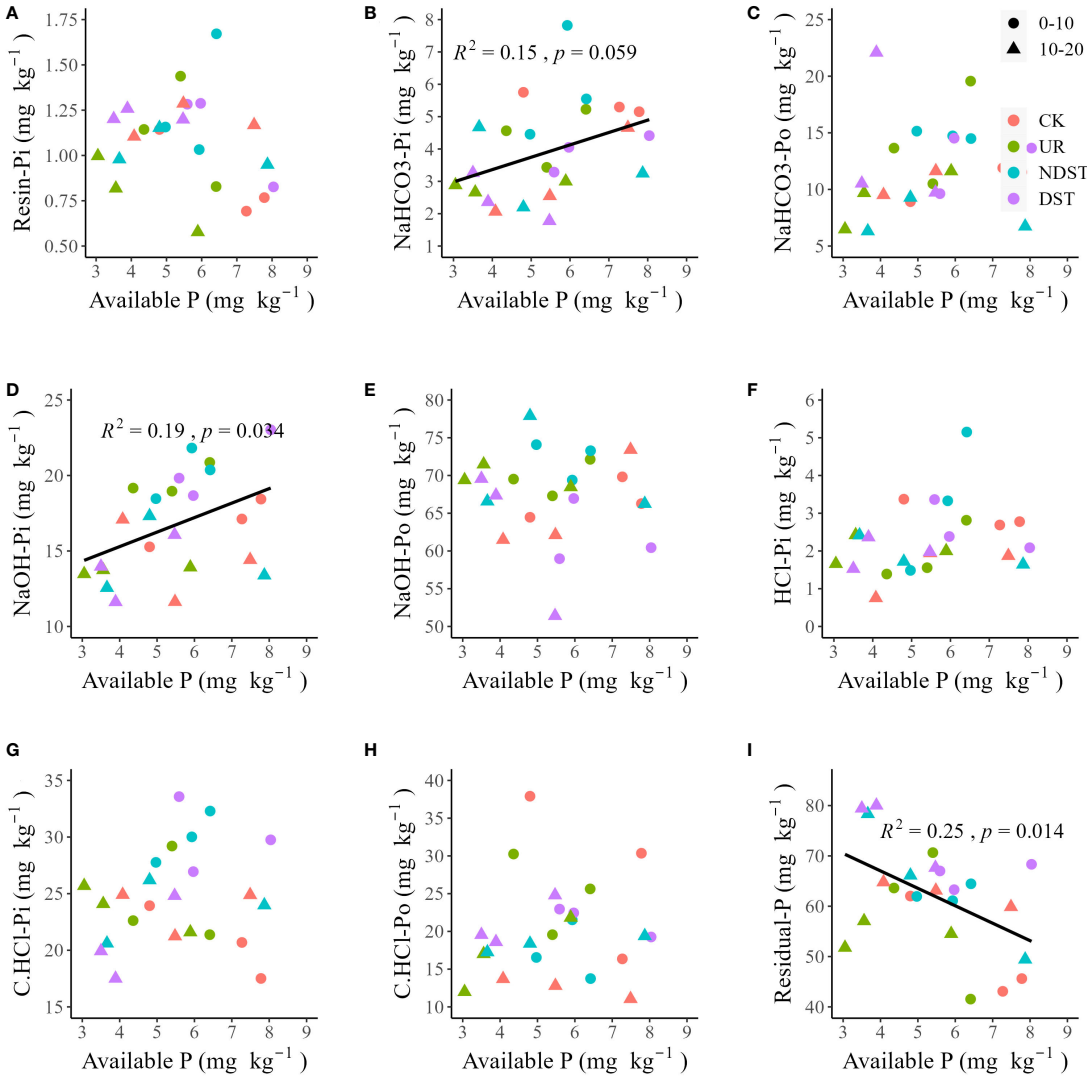
Correlations of soil available P with resin-Pi **(A)**, NaHCO_3_-Pi **(B)**, NaHCO_3_-Po **(C)**, NaOH-Pi **(D)**, NaOH-Po **(E)**, HCl-Pi **(F)**, C.HCl-Pi **(G)**, C.HCl-Po **(H)** and residual-P **(I)**. CK, control; UR, understory removal; NDST, non-dominant species thinning; DST, dominant species thinning; 0–10, 0–10 cm soil layer; 10–20, 10–20 cm soil layer; Pi, inorganic phosphorus; and Po, organic phosphorus.

**Figure 4:**
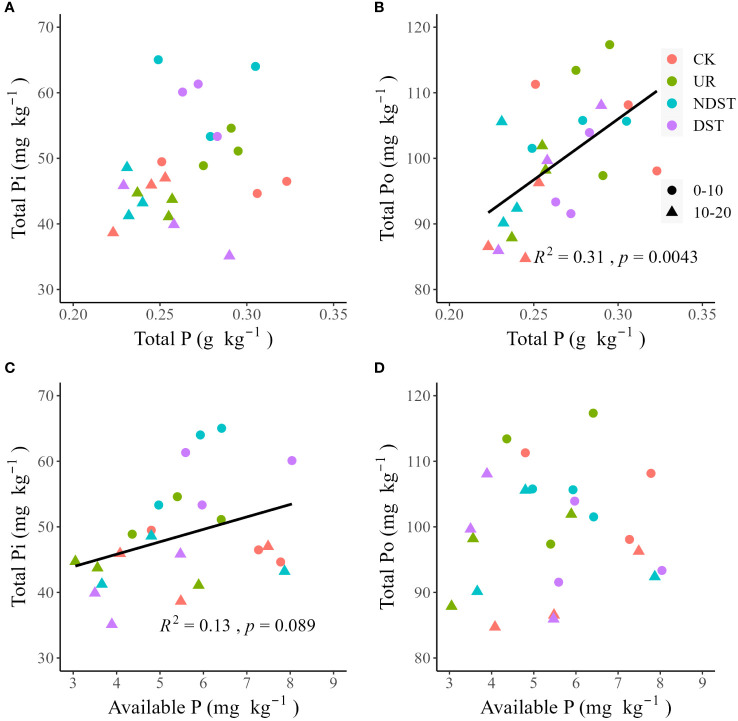
Correlations of total P with total Pi **(A)** and total Po **(B)**, as well as correlations of available P with total Pi **(C)** and total Po **(D)**. CK, control; UR, understory removal; NDST, non-dominant species thinning; DST, dominant species thinning; 0–10, 0–10 cm soil layer; 10–20, 10–20 cm soil layer; Pi, inorganic phosphorus; and Po, organic phosphorus.

Soil total P was positively related to soil NaHCO_3_-Pi (*R* ^2^ = 0.29, *p* < 0.01; **Figure 2B**), NaHCO_3_-Po (*R* ^2^ = 0.32, *p* < 0.01; **Figure 2C**) and NaOH-Pi (*R* ^2^ = 0.24, *p* < 0.05; **Figure 2D**). Soil total P was not significantly related to soil resin-Pi, NaOH-Po, HCl-Pi, C.CHCl-Pi, C.CHCl-Po and residual-P (**Figures 2A**, **E–I**). Soil available P was positively correlated with NaHCO_3_-Pi (*R* ^2^ = 0.15, with a marginally significant level of 0.059; **Figure 3B**) and soil NaOH-Pi (*R* ^2^ = 0.19, *p* < 0.05; **Figure 3D**) but was negatively correlated with soil residual-P (*R* ^2^ = 0.25, *p* < 0.05; **Figure 3I**). Soil available P was not significantly correlated with soil resin-Pi, NaHCO3-Po, NaOH-Po, HCl-Pi, C.CHCl-Pi and C.CHCl-Po (**Figures 3A, C, E–H**). Also, there were positive relationships between total P and total Po (*R* ^2^ = 0.31, *p* < 0.01; **Figure 4B**) and between available P and total Pi (*R* ^2^ = 0.13, with a marginally significant level of 0.089; **Figure 4C**). There were not significant relationships between total P and total Pi (**Figure 4A**) and between available P and total Po (**Figure 4D**). Significantly positive relationships among NaHCO_3_-Pi, NaOH-Pi, and HCl-Pi, as well as between resin-Pi and residual-P, were observed (**Supplementary Figure S1**).

## Discussions

### Forest management decreased soil P content

Our results identified that both total P and available P in UR, NDST, and DST plantations were lower than those in CK plots (**Figure 1**), which agreed with the first hypothesis but did not support the results of the previous meta-analysis (Zhou et al., 2021; Zhang et al., 2022a, 2023). In the P-deficient pine plantations (Reichert et al., 2022; Jian et al., 2022b), the rapid growth of the remaining trees after forest management (**Table 1**) accelerated the plant P uptake from soils (Rocha et al., 2019) and then decreased soil P. Moreover, high total P and available P contents in topsoils were likely related to the P biogeochemical cycle: movement of soil P from subsoil (Zhou et al., 2021) and return of P element in plant organs to the topsoil via litter, detritus, and roots decomposition (Hu et al., 2016). Unfortunately, this study did not measure the relevant data, and further confirmation is needed for these mechanisms.

Interestingly, total P (0.27–0.29 g·kg^-1^) and available P (5.39–6.62 mg·kg^-1^) of 0–10 cm after nine years were higher than those in the initial stage of forest management (0.22–0.24 g·kg^-1^ and 0.86–2.15 mg·kg^-1^, respectively; Shen et al., 2017; Lei et al., 2021). Forest management increased soil temperature (Zeng et al., 2023) and understory diversity (Wang et al., 2019), which accelerates litter decomposition (Hu et al., 2016) and promotes soil microbial biomass (Lei et al., 2021) and activity (Rocha et al., 2019), and then improved P availability in the pine plantations.

### Diverse effects of forest management on soil P fractions

Plants can absorb directly resin-P, NaHCO_3_-Pi, and NaHCO_3_-Po from soils (Hedley et al., 1982). Compared to CK plots, these soil P fractions in UR, NDST, and DST plots increased in 0–10 cm but decreased in 10–20 cm (**Table 2**), consistent with previous results (Liu et al., 2018b; Ye et al., 2018). The high affinity of humic acid for Fe and Al ions can weaken the adsorption of Fe and Al ions at mineral surfaces to Pi (Gérard, 2016), thus preventing P deposition and increasing soil P availability. Also, root exudates can decompose the moderately labile P and occluded P (Broeckling et al., 2008), and microorganisms can hydrolyze Po (Zhang et al., 2016), thereby promoting the transformation of NaOH-Po and C.HCl-Po to the active P (Fan et al., 2018; Liu et al., 2021).

As a potential P source for plant absorption, soil NaOH-Pi was higher, but soil NaOH-Po was lower in DST plots than in CK plots (**Table 2**). These results differed from previous studies where slight and moderate logging significantly reduced NaOH-Po but increased NaOH-Pi (Liu et al., 2018b; Ye et al., 2018). Likely, the understory shrubs and herbs with arbuscular mycorrhizal symbiosis in DST plots (Wang et al., 2019) promoted the secretion of phosphatase and root exudates, thereby mineralization of Po. Moreover, several studies did not observe the thinning effects on soil occluded P (Hu et al., 2016; Liu et al., 2018b; Ye et al., 2018). However, NDST and DST treatments significantly increased soil C.HCl-Pi in 0–10 cm and C.HCl-Po in 10–20 cm in the pine plantations (**Table 2**). Differences in climatic factors and soil types in various study areas are likely related to the inconsistent findings, which need further confirmation.

### The potential contribution of residual-P to available P after forest management

Forest management increased total Pi but decreased total Po of 0–10 cm (**Table 2**), supporting previous findings (Liu et al., 2018b; Ye et al., 2018) and our second hypothesis. These results suggested that forest management treatments exacerbated the P demand of plants due to high growth (**Table 1**). On the one hand, Pi is the main form of plant absorption and utilization (Hedley et al., 1982), and the soil Pi content is mainly dominated by the balancing process between different P fractions (Walker and Syers, 1976), leading to an increase in soil total Pi. On the other hand, the selected pine species are ECM, and mycorrhizal symbiotes can absorb Po fractions (Liu et al., 2018a; Mei et al., 2024), leading to a decrease in soil total Po. Residual-P accounted for approximately 24.6%-35.6% of soil P fractions (**Table 2**). It was negatively related to available P (**Figure 3I**) and positively associated with resin-Pi (**Supplementary Figure S1**). These results pointed out the potential contribution of residual-P to available P after forest management, which partially supports our third hypothesis. We also acknowledge that this finding is from correlation analysis and theoretical inference, and further confirmation of the transformation mechanism between soil P fractions is needed to determine whether plants can absorb various P fractions.

## Conclusions

Compared to plantations without management, total P and available P declined while total Pi increased in topsoils in subtropical pine plantations treated by understory removal and thinning after nine years. Selective thinning treatments promoted the accumulation of occluded P, including C.HCl-Pi and C.HCl-Po. The negative relationship between residual-P and available P and the positive relationship between residual-P and resin-Pi suggest that transforming residual-P into available P may significantly contribute to the high plant P demands due to the high growth rate after forest management.

## Data availability statement

The original contributions presented in the study are included in the article/**Supplementary Material**. Further inquiries can be directed to the corresponding author.

## Author contributions

ZJ: Formal analysis, Methodology, Writing – original draft. LZ: Conceptualization, Data curation, Writing – review & editing. LL: Conceptualization, Funding acquisition, Writing – review & editing. CL: Funding acquisition, Supervision, Writing – review & editing. YS: Investigation, Project administration, Writing – review & editing. JZ: Investigation, Writing – review & editing. WX: Conceptualization, Supervision, Writing – review & editing. M-HL: Writing – review & editing.
