# Supplementary material for: Effects of thinning and understory removal on soil phosphorus fractions in subtropical pine plantations
Source: Front Plant Sci. 2024 Jun 25;15:1416852. doi: 10.3389/fpls.2024.1416852 (PMC11231387; doi:10.3389/fpls.2024.1416852)
Supplement: Supplementary file 1 [file DataSheet_1.docx]

**Supplementary Material**

**Effects of thinning and understory removal on soil phosphorus fractions in subtropical pine plantations**

Zunji Jian^1^, Lixiong Zeng^1,2^, Lei Lei^1,2,*^, Changfu Liu^1,2^, Yafei Shen^1,2^,

Jiajia Zhang^1^, Wenfa Xiao^1,2^, Mai-He Li^3,4,5^

^1^*Key Laboratory of Forest Ecology and Environment of National Forestry and Grassland Administration, Ecology and Nature Conservation Institute, Chinese Academy of Forestry; Beijing, China*

^2^*Co-Innovation Center for Sustainable Forestry in Southern China, Nanjing Forestry University, Nanjing, China*

^3^*Forest Dynamics, Swiss Federal Institute for Forest, Snow and Landscape Research WSL, Zuercherstrasse 111, Birmensdorf CH-8903, Switzerland*

^4^*Key Laboratory of Geographical Processes and Ecological Security in Changbai Mountains, Ministry of Education, School of Geographical Sciences, Northeast Normal University, Changchun, China*

^5^*School of Life Science, Hebei University, Baoding, China*

Author's E-mail: Zunji Jian [(jianzunji2014@163.com);](mailto:(jianzunji2016@163.com);)

Lixiong Zeng [(zlxcaf@163.com);](mailto:(zlxcaf@163.com);)

Lei Lei [(cafleilei@163.com);](mailto:(cafleilei@163.com);)

Changfu Liu [(liucf898@163.com](mailto:(liucf898@163.com));

Yafei Shen [(yafeishen126@126.com);](mailto:(yafeishen126.@126.com);)

Jiajian Zhang [(zhangjiajia@caf.ac.cn](mailto:(zhangjiajia@caf.ac.cn));

Wenfa Xiao [(xiaowenf@caf.ac.cn)](mailto:(xiaowenf@caf.ac.cn));

Mai-He Li [(Maihe.li@wsl.ch](mailto:(Maihe.li@wsl.ch)).

* Corresponding author. [cafleilei@163.com](mailto:cafleilei@163.com)

**Table S1.** Results of two-way ANOVA test on soil P fractions after nine years of forest management in a pine plantation of subtropical China.

| P fraction | Treatment | | Layer | | Treatment×Layer | |
| --- | --- | --- | --- | --- | --- | --- |
|  | *F*-value | *p*-value | *F*-value | *p*-value | *F*-value | *p*-value |
| Total P | 0.403 | 0.753 | 17.449 | **<0.001** | 0.901 | 0.462 |
| Available P | 0.909 | 0.458 | 3.969 | **0.064** | 0.454 | 0.718 |
| Resin-Pi | 1.221 | 0.334 | 0.276 | 0.606 | 2.807 | 0.073 |
| NaHCO_3_-Pi | 2.459 | 0.100 | 22.638 | **<0.001** | 0.443 | 0.725 |
| NaHCO_3_-Po | 0.672 | 0.582 | 4.009 | **0.063** | 2.439 | 0.102 |
| NaOH-Pi | 0.899 | 0.463 | 43.143 | **<0.001** | 1.297 | 0.310 |
| NaOH-Po | 3.236 | 0.050 | 0.075 | 0.787 | 0.078 | 0.971 |
| HCl-Pi | 0.624 | 0.610 | 6.171 | **0.024** | **1.148** | 0.360 |
| C.HCl-Pi | 2.481 | 0.098 | 7.212 | **0.016** | **4.966** | **0.013** |
| C.HCl-Po | 0.588 | 0.632 | 7.959 | **0.012** | **3.455** | **0.042** |
| Residual-P | 3.718 | **0.033** | 1.909 | 0.186 | 1.075 | 0.388 |
| Total Pi | 3.174 | 0.053 | 44.166 | **<0.001** | 4.171 | **0.023** |
| Total Po | 0.656 | 0.591 | 8.151 | **0.012** | 1.540 | 0.243 |

Values are shows as the F-value and p-value based on the two-way ANOVA test. Bold values represent significant levels of 0.05. Pi, inorganic phosphorus; and Po, organic phosphorus.


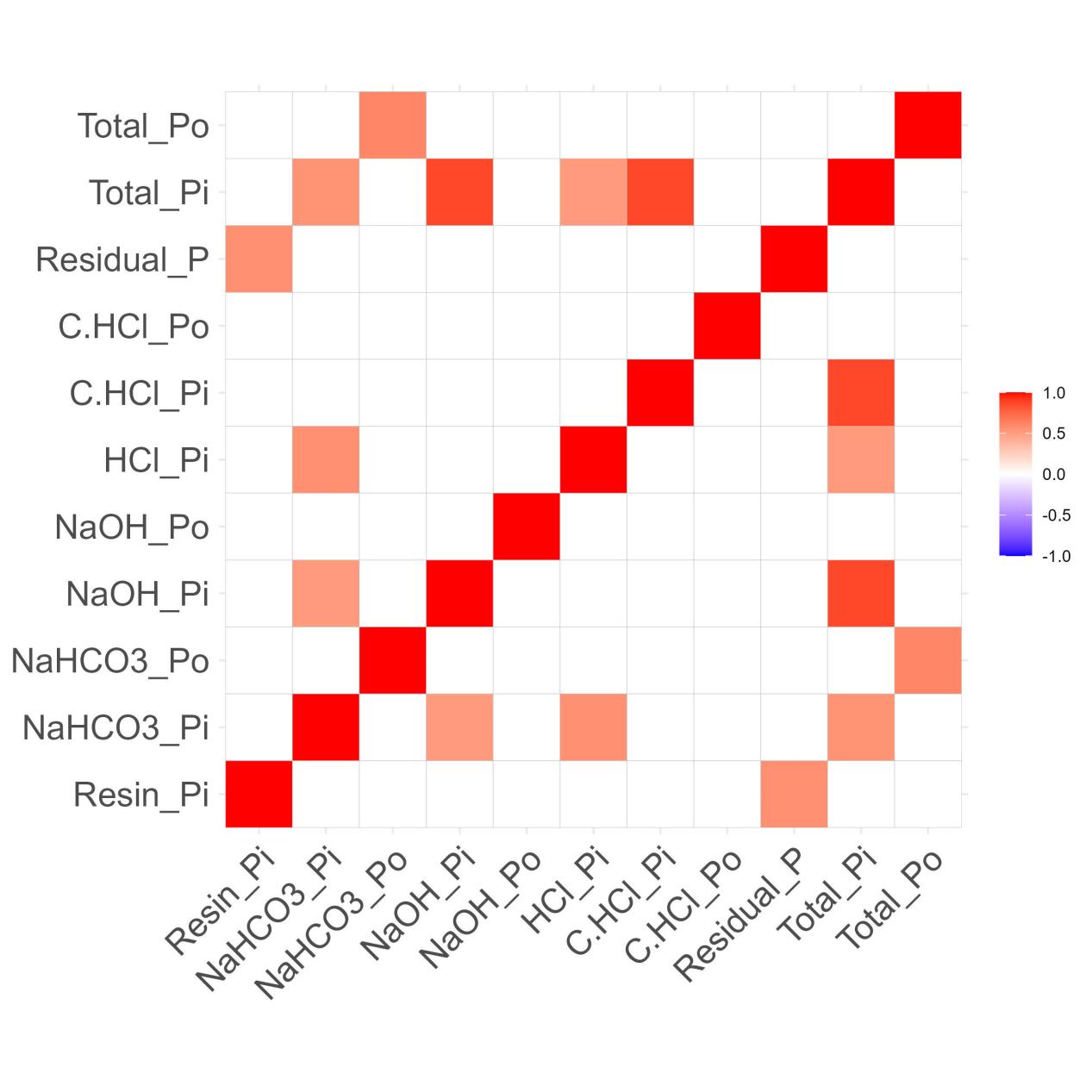


**Fig. S1.** Pearson’s correlation coefficient among soil P fractions after nine years of forest management treatments in a pine plantation of subtropical China. Pi, inorganic phosphorus; and Po, organic phosphorus.
